# Lysosomal Destabilizing Drug Siramesine and the Dual Tyrosine Kinase Inhibitor Lapatinib Induce a Synergistic Ferroptosis through Reduced Heme Oxygenase-1 (HO-1) Levels

**DOI:** 10.1155/2019/9561281

**Published:** 2019-09-17

**Authors:** Gloria E. Villalpando-Rodriguez, Anna R. Blankstein, Carmen Konzelman, Spencer B. Gibson

**Affiliations:** ^1^Research Institute in Oncology and Hematology, CancerCare Manitoba, University of Manitoba, Winnipeg, Manitoba, Canada R3E OV9; ^2^Department of Biochemistry and Medical Genetics, University of Manitoba, Winnipeg, Manitoba, Canada R3E 0J9

## Abstract

Ferroptosis is an iron-dependent type of cell death distinct from apoptosis or necrosis characterized by accumulation of reactive oxygen species. The combination of siramesine, a lysosomotropic agent, and lapatinib, a dual tyrosine kinase inhibitor (TKI), synergistically induced cell death in breast cancer cells mediated by ferroptosis. In this study, we showed that this combination of siramesine and lapatinib induces synergistic cell death in glioma cell line U87 and lung adenocarcinoma cell line A549. This cell death was characterized by the increase in iron content, reactive oxygen species (ROS) production, and lipid peroxidation accumulation after 24 hours of treatment. Moreover, iron chelator DFO and ferrostatin-1, a ferroptosis inhibitor, significantly reduced cell death. The mechanism underlying the activation of the ferroptotic pathway involves lysosomal permeabilization and increase in reactive iron levels in these cells. In addition, the downregulation of heme oxygenase-1 (HO-1) protein occurred. Overexpression of HO-1 resulted in reduction of ROS and lipid peroxidation production and cell death. Furthermore, knocking down of HO-1 combined with siramesine treatment resulted in increased cell death. Finally, we found that the inhibition of the proteasome system rescued HO-1 expression levels. Our results suggest that the induction of ferroptosis by combining a lysosomotropic agent and a tyrosine kinase inhibitor is mediated by iron release from lysosomes and HO-1 degradation by the proteasome system.

## 1. Introduction

In cancer cells, the most common types of cell death such as apoptosis are often actively inhibited, contributing to the development of drug resistance. Identifying and exploiting alternative cell death pathways are essential in overcoming or bypassing drug resistance. In glioblastoma and lung adenocarcinoma cells, drug resistance is a major obstacle in developing effective treatments. Recently, we discovered an innovative drug combination that induces a new form of cell death called ferroptosis in breast cancer cells [1].

Ferroptosis is a cell death mechanism that is morphologically, biochemically, and genetically distinct from other types of cell death. It is characterized by the iron-dependent intracellular accumulation of reactive oxygen species (ROS) and lipid peroxidation products [2] [3]. Ferroptosis inducers include erastin and sorafenib that inhibit the cystine/glutamate antiporter and RAS selective lethal 3 (RSL3) by inhibition of glutathione peroxidase 4 (GPX4). In addition, alterations in iron transport regulatory proteins such as ferroportin-1 (FPN), an iron transport protein responsible for removal of iron from cells, contribute to ferroptosis. Ferroptosis can be inhibited by preventing the accumulation of ROS from lipid peroxidation using ferrostatin-1 (Fer-1) or by binding free iron in the cell using chelators like deferoxamine (DFO) [4].

Regulators of ferroptosis include the transcription factor nuclear factor erythroid 2 p45-related factor 2 (Nrf2) [4–6]. Nrf2 acts as a key regulator of antioxidant response in particular by inducing the expression of heme oxygenase-1 (HO-1). HO-1 is known to be overexpressed in cancer cells where it exerts a strong antioxidant and antiapoptotic effect favoring cancer cell growth and resistance to therapy [7–10].

HO-1 is an enzyme that degrades heme into ferrous iron, carbon monoxide, and biliverdin which is then reduced to bilirubin by biliverdin reductase. The antioxidant activity that is attributed to HO-1 comes from its by-products biliverdin and bilirubin. Indeed, studies in vascular endothelial cells showed a protective effect of bilirubin. Moreover, it was found that knocking down biliverdin reductase attenuated the hypoxia-induced resistance in glioblastoma and reverses multidrug resistance in leukemic cells [11–15].

Previous studies in our laboratory showed that the combination of a lysosomotropic agent siramesine and lapatinib, a tyrosine kinase inhibitor, synergistically induced cell death accompanied by increased ROS production and lipid peroxidation in breast cancer cell lines. The cell death observed with the combination was blocked by Fer-1 and DFO, suggesting that cell death was occurring via ferroptosis [1]. Lysosomotropic agents such as siramesine are weak bases able to diffuse across the lysosomal membrane; when they reach this compartment, they become protonated and can no longer pass through the lysosomal membrane, thus accumulating within the lysosome. This accumulation destabilizes the lysosomal membrane causing the leakage of its content into the cytosol [16, 17]. Lysosomes contain a major portion of redox-active iron due to degradation of ferruginous material [18–20]. Lapatinib is a tyrosine kinase inhibitor of epidermal growth factor receptor (EGFR) and Erb2 (Her2) tyrosine kinases. Studies *in vitro* showed that lapatinib inhibited proliferation of ErbB2 and EGFR overexpressing cancer cells and induced apoptosis mediated in part by ROS [21, 22]. Whether the combination of siramesine and lapatinib gives the best synergistic cell death response in glioblastoma and lung cancer cell lines is unknown, and whether the mechanism of inducing ferroptosis is similar to breast cancer cells is unclear.

In this study, we investigated the effect of lysosome disruptors and tyrosine kinase inhibitor treatment in glioblastoma and lung cancer cell lines. We found that siramesine and lapatinib gave the best combination index and this combination induced ferroptosis through iron-mediated ROS and downregulation of HO-1 levels.

## 2. Materials and Methods

### 2.1. Reagents and Antibodies

Siramesine (Prod. No. SML0976), desipramine (Prod. No. D3900), trypan blue solution (Prod. No. T8154), tafenoquine (Prod. No. SML0396), clemastine (Prod. No. SML0445), loratadine (Prod. No. L9664), desloratadine (Prod. No. D1069), erastin (Prod. No. E7781), FeCl_3_ (Prod. No. 157740), deferoxamine (Prod. No. D9533), ferrostatin-1 (Prod. No. SML0583), bilirubin (Prod. No. B4126-1G), Prussian blue soluble (Prod. No. 03899), and MG132 (Prod. No. M7449-200UL) are from Sigma-Aldrich; gefitinib (Prod. No. 2030) from Cedarlane Labs, sorafenib (Prod. No. S-8599) and lapatinib (Prod. No. L-4899) from LC Labs; and cobalt protoporphyrin IX chloride (Prod. No. ALX430-076) from Enzo. The siRNA against HO-1 (sc-35554) was purchased from Santa Cruz Biotechnology Inc. (Dallas, TX, USA). Primary antibodies anti-Nrf2 (#12721), anti-ubiquitin (#3936), anti-TfR1 (#13113S), anti-DMT1 (#15083S), and anti-ferritin (#4393S) were purchased from Cell Signaling Technology (Beverly, MA, USA); anti-transferrin (ab9538) and anti-HO-1 (ab68477) from Abcam (Cambridge, UK; anti-actin (Prod. No. A3853) from Sigma-Aldrich; and anti-ferroportin (NBP1-21502) from Novus Biologicals. Secondary antibodies goat anti-rabbit IgG (H+L)-HRP conjugate (Cat. No. 170-6515) and goat anti-mouse IgG (H+L)-HRP conjugate (Cat. No. 170-6516) were obtained from Bio-Rad Laboratories (Hercules, CA, USA) (ab9538). LysoTracker (Cat.: L-7526), H2DCF Cat.: D-399), dihydroethidium (Cat.: D-1168), and CODIPY-C11 (Cat.: D-3861) are from Life Technologies (Thermo Fisher Scientific, Waltham, MA, USA).

### 2.2. Cell Culture

The glioblastoma U87 and the lung adenocarcioma A549 cell lines were grown in Dulbecco's modified Eagle medium (DMEM, high glucose; GIBCO, Cat. 10565-018, Life Technologies) supplemented with 100 units of penicillin per ml plus 100 *μ*g of streptomycin per ml (Cat. 10378016, Life Technologies) and 10% fetal bovine serum, in a humidified 5% CO_2_, 37°C incubator. Cells were treated for various times in the absence and presence of a chemical inhibitor.

### 2.3. Measurement of Cell Death by Flow Cytometry

Cell death was measured by staining with trypan blue to detect the plasma membrane integrity through flow cytometry as described previously [23]. Briefly, trypan blue is excluded from live cells but penetrates into a dead cell giving a red fluorescent signal that can be quantified by flow cytometry.

### 2.4. Measurement of ROS by Flow Cytometry

ROS generation was determined by flow cytometry with dihydroethidium (DHE D-1168). DHE is oxidized by ROS into 2-hydroxyethidium (2-HE) (emission at 605 nm) and fluoresces red. The samples were collected and stained with 5 *μ*M DHE and then incubated in the dark in a water bath at 37°C for 15 min. The cell suspension was then transferred to a 5 ml FACS tube and analyzed on a flow cytometer within 10 min using Novocyte software (ACEA Biosciences Inc.).

### 2.5. Measurement of Lipid Peroxidation by Flow Cytometry

Lipid peroxidation was determined by flow cytometry with C11-BODIPY 581/591 (D-3861). Oxidation of a component of the C11-BODIPY fluorophore shifts the fluorescence emission from red to green, and a change in the ratio of green to red fluorescence was used as an indicator of an increase in lipid peroxidation. The samples were collected and stained with 1 *μ*M C11-BODIPY and then incubated in the dark in a water bath at 37°C for 15 min. The cell suspension was then transferred to a 5 ml FACS tube and analyzed on a flow cytometer within 10 min using Novocyte software (ACEA Biosciences Inc.).

### 2.6. Flow Cytometry for LMP Detection

The LMP of the treated cells was measured using LysoTracker Green DND-26 (Thermo Fisher Scientific) staining. LysoTracker dye selectively accumulates in cellular compartments with low internal pH where it exhibits fluorescence. A decrease in this fluorescence is interpreted as LMP. In brief, the culture medium was aspirated after treatment and LysoTracker 50 nM in culture medium was added to the cells. After 15 min of incubation at 37°C, the cells were collected and the cell suspension was then transferred to a 5 ml FACS tube and analyzed on a flow cytometer within 10 min using Novocyte software (ACEA Biosciences Inc.).

### 2.7. Transfection of siRNA

The transfection of cells with siRNA and plasmid was done as described in our previous studies [24].

### 2.8. Nuclear Fractionation

Cells were collected, and the nuclear and cytoplasmic fractions were obtained using the NE-PER Nuclear and Cytoplasmic Extraction kit (Thermo Fisher Scientific) according to the manufacturer's instructions. Fractions were analyzed by Western blot as mentioned below.

### 2.9. Western Blot Analysis

Cell lysates were collected at the indicated times in 1% NP-40 lysis buffer with a complete protease inhibitor tablet (Roche, Basel, Switzerland) and phosphatase inhibitor cocktail 2 and 3 (Sigma-Aldrich). Protein levels were quantified with a Pierce BCA kit (Thermo Fisher Scientific) according to the manufacturer's instructions. Samples were run on 10% polyacrylamide gels and transferred onto nitrocellulose membranes (Bio-Rad, Hercules, CA, USA), blocked in 5% milk in TBS-T as per the antibody manufacturer's suggestions. Secondary antibodies were goat anti-rabbit-HRP or anti-mouse-HRP (Bio-Rad). Detection of protein was with Pierce ECL or Pierce SuperSignal Pico (Thermo Fisher Scientific) reagents.

### 2.10. Prussian Blue Staining

Prussian blue staining was used to detect the presence of iron oxide nanoparticles. The cells were fixed in 4% paraformaldehyde for 30 min, washed 3 times with PBS, incubated for 30 min with Prussian blue (10 mg/ml), and then rewashed three times with PBS. Labeled cells were examined under a light microscope to determine intracellular iron oxide distribution.

### 2.11. Calcein Assay

Labile iron pool was determined by flow cytometry with calcein-AM (Thermo Scientific). When calcein enters the cell, it binds iron, which quenches its fluorescent signal. Upon addition of deferiprone, the iron becomes preferentially bound to deferiprone and the fluorescent signal of calcein is no longer quenched. The difference in fluorescent signal between the deferiprone-treated and untreated cells was used as an indirect measure of the labile iron pool. The samples were collected and incubated with 1 *μ*M calcein-AM in the dark in a water bath at 37°C for 15 min; then, the cells were washed and treated with 100 *μ*M deferiprone (Sigma-Aldrich), an iron chelator, or left untreated, for one hour at 37°C in the dark. The cell suspension was then transferred to a 5 ml FACS tube and analyzed on a flow cytometer within 10 min using Novocyte software (ACEA Biosciences Inc.).

### 2.12. Assessment of Drug Interaction

Interaction between siramesine and lapatinib was assessed according to the method of Chou and Talalay [25], where the combination index (CI) is defined as follows:

(1) $$\begin{array}{c} \text{CI}=\frac{{\text{ds}}_{\text{x}}}{{\text{Ds}}_{\text{x}}}+\frac{{\text{dl}}_{\text{x}}}{{\text{Dl}}_{\text{x}}}, \end{array}$$

where ds_x_ and dl_x_ are doses of siramesine and lapatinib, respectively, required to produce a given reduction in cell viability when given in combination, and Ds_x_ and Dl_x_ are doses of siramesine and lapatinib, respectively, required to produce the same effect in single-agent treatments. CI < 1, =1, and >1 are interpreted as synergy, additivity, and antagonism, respectively.

### 2.13. Statistical Analysis

All data were generated with at least three independent experiments. Each experiment in the cell death analysis was carried out by 3–4 replicates. The data were represented as means ± S.D. (*n* ≥ 3). Student's *t*-test or one-way ANOVA was performed for statistical analysis with *P* > 0.05 being considered as statistical significance.

## 3. Results

### 3.1. Lapatinib and Siramesine Induce Synergistic Cell Death in Lung Adenocarcinoma and Glioblastoma Cells to a Greater Extent than Other Lysosomotropic Agents and Tyrosine Kinase Inhibitors

**Figure 1:**
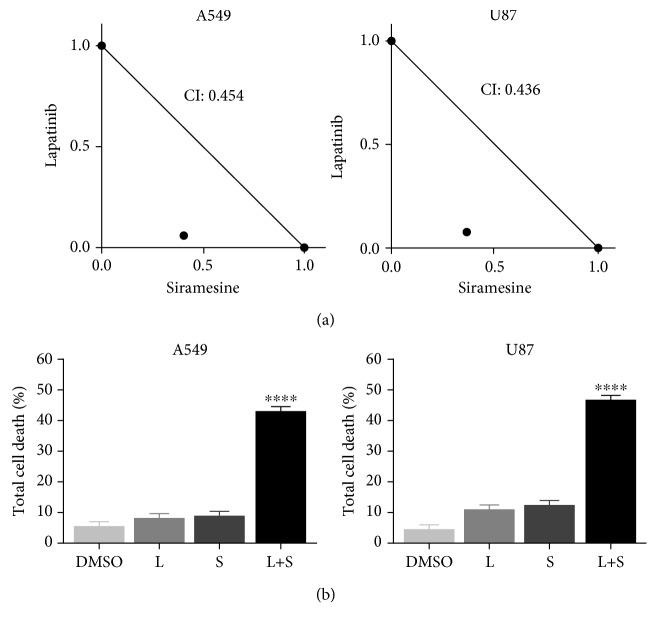
Siramesine and lapatinib induce synergistic cell death. (a) A549 and U87 were treated for 24 hours with 0.5 *μ*M lapatinib and 10 *μ*M siramesine. Drug-drug interactions were assessed according to the combination index (CI) model. Isobolograms demonstrate the effects of the combination. (b) A549 and U87 were treated for 24 hours with DMSO (D), 0.5 *μ*M lapatinib (L), 10 *μ*M siramesine (S), or the combination of lapatinib+siramesine (L+S). Cell death was quantified by trypan blue exclusion assay using flow cytometry. Standard error represents four independent experiments (*n* = 4). ^∗∗∗∗^ represents statistical significance of *P* < 0.0001.

**Figure 2:**
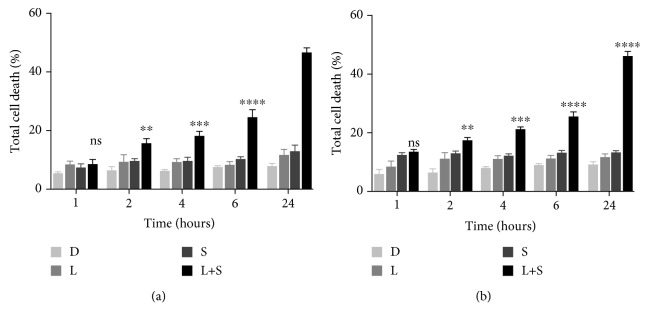
Time course of siramesine and lapatinib-induced cell death. (a) A549 and (b) U87 cells were treated with DMSO (D), lapatinib (L) (0.5 *μ*M), siramesine (S) (10 *μ*M), or the combination of lapatinib+siramesine (L+S) for 1, 2, 4, 6, and 24 hours. Cell death was measured by trypan blue staining and quantified by flow cytometry. Standard error represents three independent experiments (*n* = 3). ns: not significant. Asterisks represent statistical significance: ^∗∗^*P* < 0.01, ^∗∗∗^*P* < 0.001, and ^∗∗∗∗^*P* < 0.0001.

We first determine if siramesine alone was capable to induce cell death, for these A549 (lung adenocarcinoma) or U87 (glioblastoma) cells were treated with different concentrations of siramesine. Results showed that in order to achieve 50% cell death, the dose of siramesine needed was relatively high (≈25 *μ*M) (Fig. [Supplementary-material supplementary-material-1]). Since previous studies showed that the combination of siramesine (with lower concentrations) and lapatinib was capable to induce synergistic cell death in breast cancer cell lines, we tested different combinations of tyrosine kinase inhibitors (TKIs) and lysosomotropic agents. To determine the best combination, in A549 and U87 cells, we treated cells with different lysosomal disruptors (antidepressants desipramine and siramesine, antihistamines clemastine and loratadine, and antimalarial tafenoquine) in combination with TKIs (lapatinib, gefitinib, and sorafenib). The lowest combination index (CI) was found when cells were treated with siramesine and lapatinib with a CI = 0.454 in A549 cells and CI = 0.438 in U87 cells (Figure 1(a)). We found that after 24-hour treatment, 10 *μ*M siramesine combined with 5 *μ*M lapatinib induced a synergistic cell death (>45%) whereas each of these drugs alone did not induce cell death significantly (<20%) when compared with the vehicle control (Figure 1(b)). During a 24-hour time course, the siramesine and lapatinib treatment increased cell death over this time course whereas the drugs alone failed to induce cell death in A549 (Figure 2(a)) as well as in U87 (Figure 2(b)). The other combinations with lysosomal disruptors mentioned above showed synergistic cell death when combined with lapatinib in both A549 and U87 cells but to a lesser extent than siramesine (Fig. [Supplementary-material supplementary-material-1]). Gefitinib is a tyrosine kinase inhibitor targeting EGFR similar to lapatinib but failed to show synergy with lysosome disruptors including siramesine in both A549 and U87 cells with CI above 1 (Fig. [Supplementary-material supplementary-material-1]). In contrast, sorafenib, a broad tyrosine kinase inhibitor, showed synergy with siramesine in both A549 and U87 cells but lapatinib still showed higher CI in these cells (Fig. [Supplementary-material supplementary-material-1]). Thus, we used siramesine and lapatinib treatment in these cells to determine the mechanism of this synergy.

### 3.2. Lapatinib and Siramesine Treatment Induced Ferroptosis

**Figure 3:**
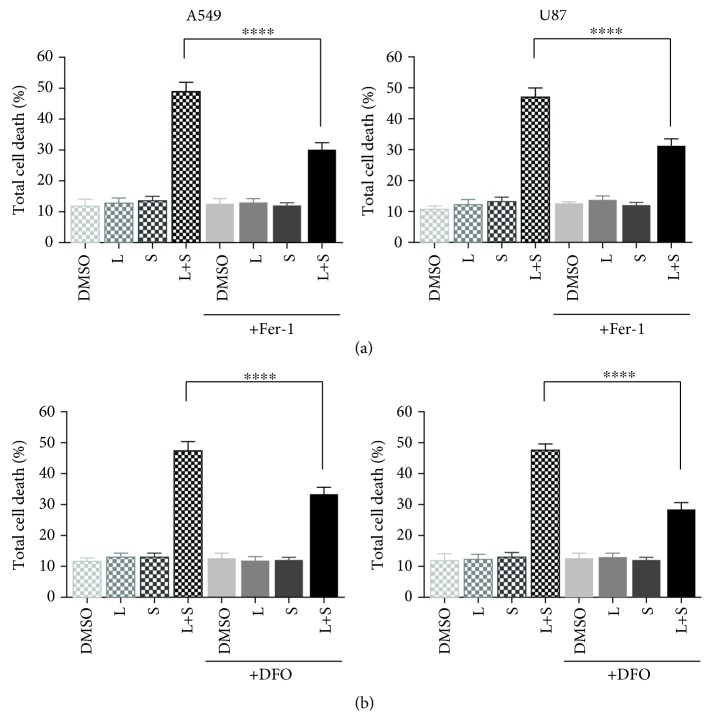
Ferrostatin-1 and DFO partially inhibits lapatinib and siramesine cell death. (a) A549 and U87 cells were pretreated with 10 *μ*M Fer-1 or (b) 200 *μ*M DFO for one hour before treatment with DMSO (D), lapatinib (L), siramesine (S), or the combination of lapatinib and siramesine (L+S) for 24 hours. Cell death was quantified by trypan blue exclusion assay using flow cytometry. Standard error represents three independent experiments (*n* = 3). ∗∗∗∗ represents statistical significance of *P* < 0.0001.

Previous studies showed that the combination of siramesine and lapatinib synergistically induce cell death in breast cancer cell lines through ferroptosis. To determine if lapatinib and siramesine-induced cell death in A459 and U87 cells was occurring through ferroptosis, the cell lines were pretreated with 10 *μ*M ferrostatin-1 (Fer-1), a potent ferroptosis inhibitor, or 200 *μ*M deferoxamine (DFO), an iron chelator; then, cells were treated with DMSO, 5 *μ*M lapatinib and 10 *μ*M siramesine alone, and in combination for 24 hours. Fer-1 showed a reduction in cell death after siramesine and lapatinib treatment, 30% cell death for the A549 cells and 32% for the U87 cells compared to 49% and 48% cell death without Fer-1 (Figure 3(a)). Similarly, DFO treatment also decreased siramesine and lapatinib-induced cell death from 48% to 30% in A549 cells and from 49% to 28% in U87 cells (Figure 3(b)). This indicates that similar to breast cancer cells, A549 and U87 cells undergo ferroptosis after siramesine and lapatinib treatment.

### 3.3. Combinational Treatment of Siramesine and Lapatinib Induces Increase in Reactive Iron and Reactive Oxygen Species

**Figure 4:**
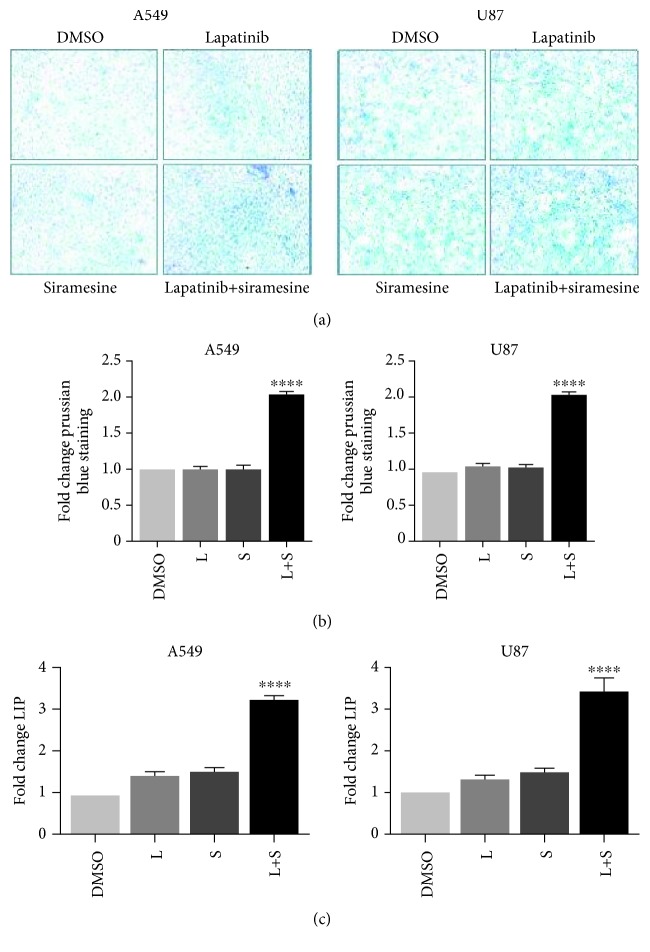
Treatment of A549 and U87 cells with the combination of siramesine and lapatinib increases the intracellular labile iron pool (LIP). (a) Prussian blue staining for intracellular iron was performed and evaluated by light microscopy after 12 hours of incubation with DMSO, lapatinib (L), siramesine (S), or with the combination of lapatinib+siramesine (L+S). Representative images from 3 separate experiments. (b) Quantitative image analysis of Prussian blue staining. Standard error represents three independent experiments (*n* = 3). ∗∗∗∗ represents statistical significance of *P* < 0.0001. (c) Calcein fluorescence assays. A549 and U87 cells were stained with calcein-AM (1 *μ*M) after 12 hours of incubation with DMSO, lapatinib (L), siramesine (S), or with the combination of lapatinib+siramesine (L+S). Fluorescence signals were measured by flow cytometry. Standard error represents three independent experiments (*n* = 3). ∗∗∗∗ represents statistical significance of *P* < 0.0001.

**Figure 5:**
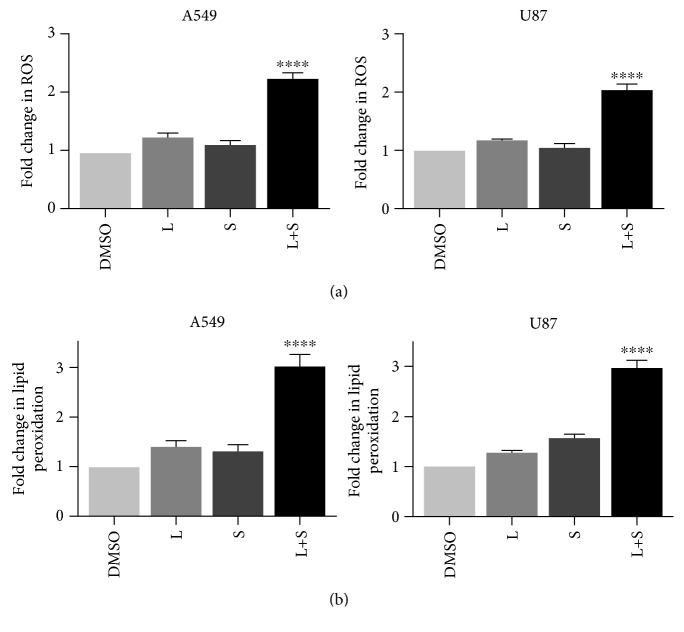
Lapatinib and siramesine combination increases ROS and lipid peroxidation. A549 and U87 cells were treated with DMSO (D), 0.5 *μ*M lapatinib (L), 10 *μ*M siramesine (S), or the combination of lapatinib+siramesine (L+S). (a) Reactive oxygen species production was measured by DHE staining. A549 and U87 cells were stained with DHE (10 *μ*M), after 24 hours of treatment; fluorescence signals were measured by flow cytometry. Standard error represents three independent experiments (*n* = 3). ∗∗∗∗ represents statistical significance of *P* < 0.0001. (b) Lipid peroxidation was measured with C11BODIPY (1 *μ*M), and after 24 hours, both red and green C11-BODIPY fluorescence were measured by flow cytometry. The change in the ratio of green to red fluorescence was used to indicate an increase in lipid peroxidation. Standard error represents three independent experiments (*n* = 3). ∗∗∗∗ represents statistical significance of *P* < 0.0001.

Ferroptosis is characterized by its dependence on iron, the accumulation of reactive oxygen species (ROS), and lipid peroxidation products. To determine if lapatinib and siramesine treatment induces ROS and lipid peroxidation, A459 and U87 cells were treated with DMSO, 5 *μ*M lapatinib and 10 *μ*M siramesine alone, and in combination; then, the labile iron pool, the ROS, and the lipid peroxidation production was measured. Our results show that the combination of siramesine and lapatinib produced a twofold increase in Prussian blue staining for intracellular iron (Figures 4(a)–4(b)) and a threefold increase in the intracellular labile iron pool (Figure 4(c)). Indeed, when we added FeCl_3_ to cells, this increased cell death in cells treated with siramesine and lapatinib (Fig. [Supplementary-material supplementary-material-1]). We also found a threefold increase in ROS as measured by DHE staining (Figure 5(a)) and twofold increase in lipid peroxidation (Figure 5(b)) compared to DMSO or either drug alone. This is consistent with the induction of ferroptosis.

**Figure 6:**
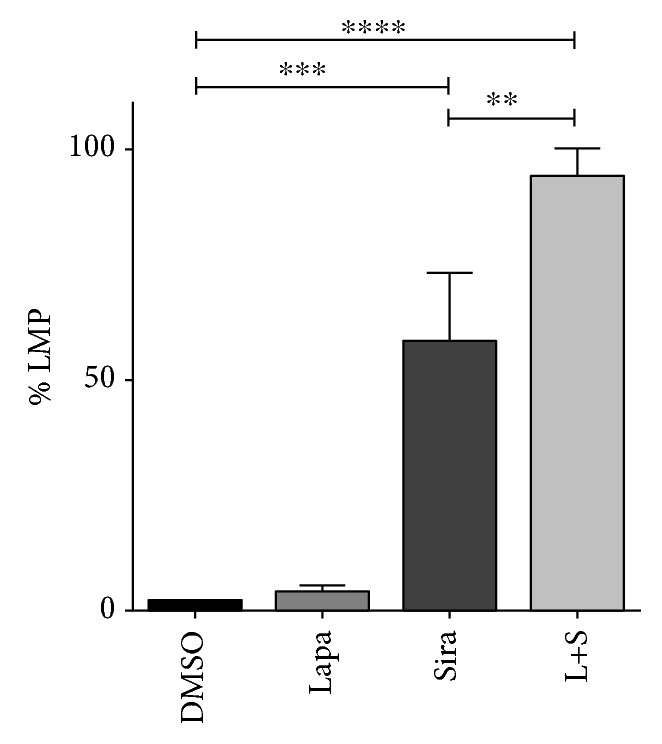
Siramesine and the combination with lapatinib induce lysosomal membrane permeabilization (LMP). U87 cells were treated with DMSO, 0.5 *μ*M lapatinib (Lapa), 10 *μ*M siramesine (Sira), or the combination of lapatinib+siramesine (L+S). After four hours of treatment, cells were incubated with 50 nM LysoTracker green for 15 min. LMP was related with the loss of fluorescence intensity of each condition when compared with the vehicle control fluorescence intensity. Standard error represents three independent experiments (*n* = 3). ∗∗∗∗ represents statistical significance of *P* < 0.0001.

Siramesine has been shown to induce lysosome membrane permeabilization (LMP) in several cancer cells including breast cancer cells. To confirm that siramesine was inducing LMP in U87 cells, cells were treated alone with lapatinib or siramesine and in combination and determined LMP as described in Material and Methods. It was found that siramesine alone induced 58% LMP and the combination accentuated this effect showing 93% LMP whereas vehicle control DMSO and lapatinib showed 2% and 4% LMP, respectively (Figure 6). Thus, similar to breast cancer cells, siramesine induced LMP in U87 cells. In order to explain the absence of LIP and cell death increase even though siramesine alone did increase LMP, we evaluate the effect of a higher concentration of siramesine and found an increase of LMP, cell death, and LIP at 50 *μ*M in the same proportions of the lapatinib-siramesine combination (Fig. [Supplementary-material supplementary-material-1]).

To further characterize ferroptosis, we found that 15 *μ*M erastin induced ferroptosis in these cells that are blocked by Fer-1 and DFO, but cell death was not observed until after 48 hours of treatment (Fig. [Supplementary-material supplementary-material-1]). We also analyzed GSH activity in U87 cells, and we found a decrease of activity in siramesine compared with vehicle control but was not further decreased following lapatinib and siramesine treatment (data not shown). Another inducer of ferroptosis is the inhibition of an iron transport system allowing for increased reactive iron in the cell. We previously found that ferroportin-1 (FPN) protein expression that exports iron out of cells is reduced upon treatment with siramesine and lapatinib in breast cancer cells and overexpression of FPN blocks cell death [1]. In A459 and U87 cells, we found that expression of iron transport proteins transferrin receptor 1 and divalent metal transporter 1 increased slightly (less than 2-fold) following siramesine and lapatinib treatment whereas transferrin, ferritin, and FPN expression fail to significantly increase (Fig. [Supplementary-material supplementary-material-1] and [Supplementary-material supplementary-material-1]) suggesting that the mechanism of ferroptosis differs from breast cancer cells.

### 3.4. The Combination of Lapatinib and Siramesine Decreases Heme Oxygenase-1 Protein Expression

**Figure 7:**
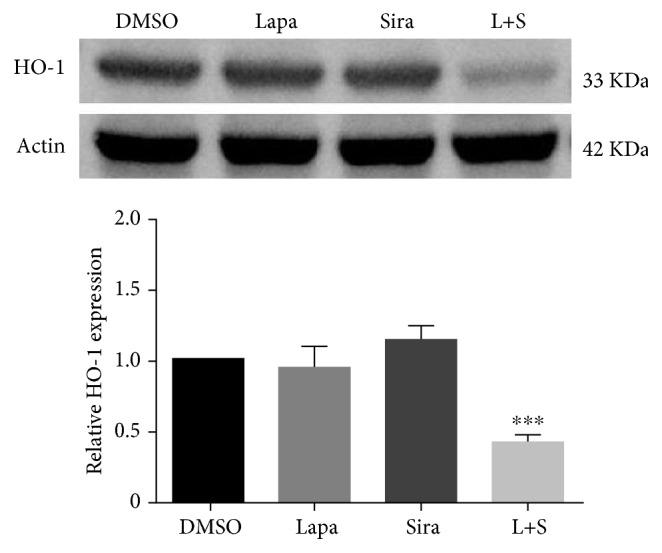
The combination of lapatinib with siramesine decreases HO-1 expression. U87 cells were treated with DMSO, 0.5 *μ*M lapatinib (Lapa), 10 *μ*M siramesine (Sira), or the combination of lapatinib+siramesine (L+S). After 24 hours of treatment, cells were lysed, Western blot determination of HO-1 was performed, and actin was used as a loading control. Densitometry was calculated using ImageJ. Standard error represents three independent experiments (*n* = 3). ∗∗∗ represents statistical significance of *P* < 0.0005.

**Figure 8:**
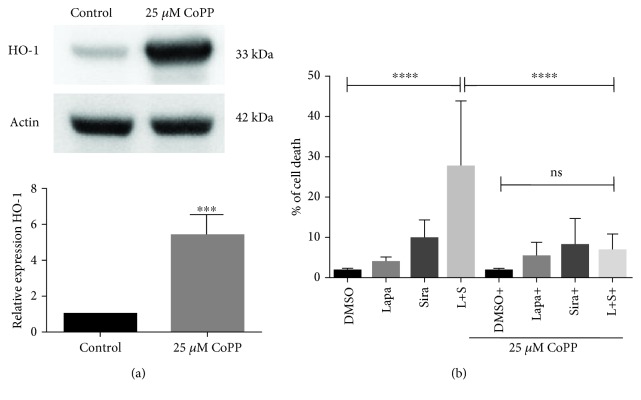
Overexpression of HO-1 inhibits lapatinib and siramesine cell death. (a) U87 cells were treated with 25 *μ*M cobalt protoporphyrin (CoPP) for 24 hours; then, cells were lysed, Western blot determination of HO-1 was performed, and actin was used as a loading control. Densitometry was calculated using ImageJ. Standard error represents three independent experiments (*n* = 3). ∗∗∗ represents statistical significance of *P* < 0.0001. (b) U87 cells were pretreated with 25 *μ*M CoPP for one hour before treatment with DMSO, 0.5 *μ*M lapatinib (Lapa), 10 *μ*M siramesine (Sira), or the combination of lapatinib+siramesine (L+S) for 24 hours. Cell death was quantified by trypan blue exclusion assay using flow cytometry. Standard error represents three independent experiments (*n* = 3). ∗∗∗∗ represents statistical significance of *P* < 0.0001; ns: not significant.

Iron-mediated ROS production is a key regulatory factor in ferroptosis. It has been shown that heme oxygenase-1 (HO-1) provides an antioxidant survival mechanism in cancer cells. U87 cells were treated with DMSO, lapatinib and siramesine alone, and in combination and the levels of HO-1 protein were determined. It was found that neither lapatinib nor siramesine alone had an effect on HO-1 expression when compared with the vehicle control; however, the combination showed a 53% decrease in protein levels (Figure 7). In addition, we found that protein levels of HO-1 were decreased with the lapatinib-siramesine combination in another glioma cell line, U373 (Fig. [Supplementary-material supplementary-material-1]) but not in A459 cells (data not shown). To determine if the decrease in HO-1 expression is responsible for at least part of the cell death induced by the lapatinib and siramesine combination, we overexpress HO-1 in U87 cells using cobalt protoporphyrin chloride (CoPP), a well-known inducer of HO-1. It was found that 25 *μ*M CoPP effectively increases HO-1 expression in U87 cells (Figure 8(a)) and that cotreatment with lapatinib and siramesine combination significantly decreases cell death when compared with the combination alone (Figure 8(b)).

**Figure 9:**
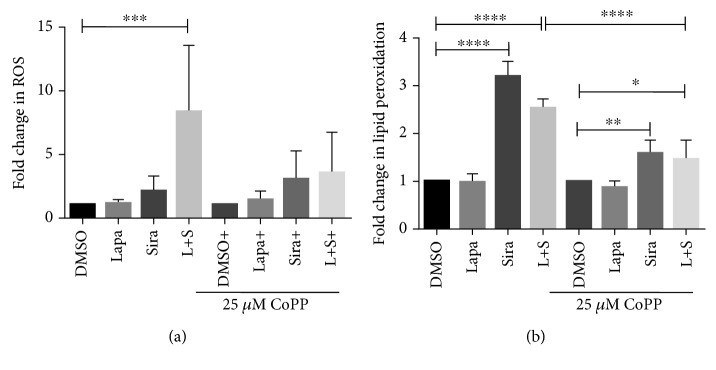
Overexpression of HO-1 protects from oxidative stress. U87 cells were pretreated with 25 *μ*M cobalt protoporphyrin (CoPP) for one hour before treatment with DMSO, 0.5 *μ*M lapatinib (Lapa), 10 *μ*M siramesine (Sira), or the combination of lapatinib+siramesine (L + S) for 24 hours. (a) Reactive oxygen species production was measured by DHE staining. Cells were stained with DHE (10 *μ*M) after treatment, and fluorescence signals were measured by flow cytometry. Standard error represents three independent experiments (*n* = 3). ∗∗∗ represents statistical significance of *P* < 0.0005. (b) Lipid peroxidation was measured with C11BODIPY (1 *μ*M), and after treatment, both red and green C11-BODIPY fluorescence were measured by flow cytometry. The change in the ratio of green to red fluorescence was used to indicate an increase in lipid peroxidation. Standard error represents three independent experiments (*n* = 3). ∗∗∗∗ represents statistical significance of *P* < 0.0001.

Because the cell death induced by lapatinib and siramesine combination is associated with an increase in ROS production and the accumulation of lipid peroxidation products, we investigate if the protective effect of HO-1 overexpression by CoPP was also associated with ROS and lipid peroxidation. For this, U87 cells were treated with DMSO, lapatinib and siramesine alone, and in combination, in the presence or absence of CoPP. It was found that pretreatment with CoPP decreases ROS production in cells treated with the combination when compared with the cells treated only with lapatinib and siramesine (Figure 9(a)). Moreover, U87 cells treated with lapatinib and siramesine in the presence of CoPP showed 1.7-fold decrease in lipid peroxidation products when compared with the combination in CoPP absence (Figure 9(b)). Thus, increased HO-1 mediated by CoPP contributes to antioxidant responses correlating to a decrease in cell death following lapatinib and siramesine treatment.

### 3.5. HO-1 By-Product Bilirubin Protects Cells

**Figure 10:**
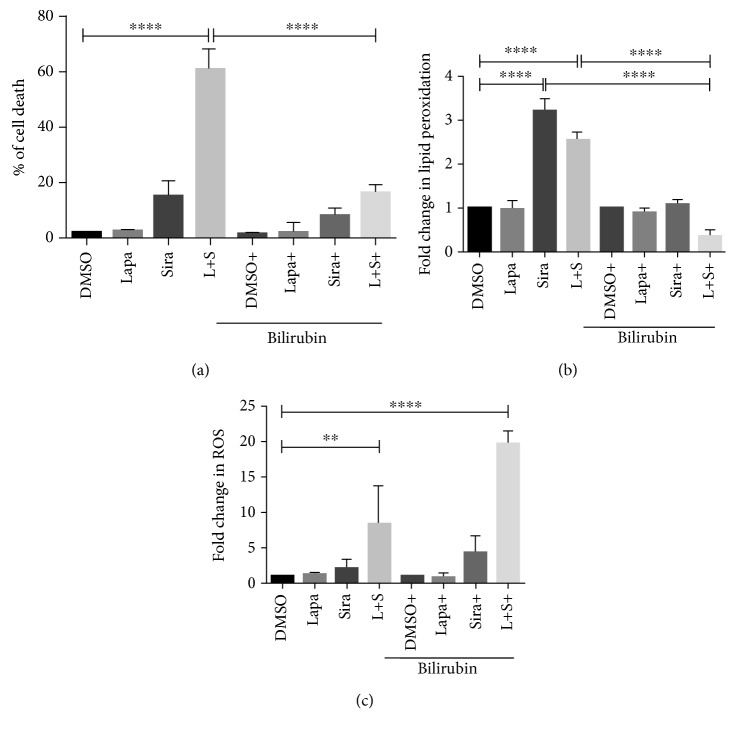
Bilirubin protects from cell death. U87 cells were pretreated with 100 nM bilirubin for one hour before treatment with DMSO, 0.5 *μ*M lapatinib (Lapa), 10 *μ*M siramesine (Sira), or the combination of lapatinib+siramesine (L+S) for 24 hours. (a) Cell death was quantified by trypan blue exclusion assay using flow cytometry. (b) Lipid peroxidation was measured with C11BODIPY (1 *μ*M), and after 24 hours, both red and green C11-BODIPY fluorescence were measured by flow cytometry. The change in the ratio of green to red fluorescence was used to indicate an increase in lipid peroxidation. (c) Reactive oxygen species production was measured by DHE staining. U87 cells were stained with DHE (10 *μ*M); after 24 hours of treatment, fluorescence signals were measured by flow cytometry. Standard error represents three independent experiments (*n* = 3). ∗∗∗∗ represents statistical significance of *P* < 0.0001.

The protective effects of HO-1 are in part accredited to its by-product bilirubin. Bilirubin has antioxidant properties protecting cells from apoptosis. We then determine whether bilirubin could confer protection against the effects of the lapatinib and siramesine treatment. U87 cells were pretreated with 100 nM bilirubin and then treated with lapatinib and siramesine alone and in combination. Results showed that only 17% cells died when pretreated with bilirubin compared to the 62% cell death induced by the combination alone (Figure 10(a)); moreover, bilirubin significantly decrease lipid peroxidation (Figure 10(b)) but fail to protect against ROS (Figure 10(c)). Thus, bilirubin is sufficient to block lapatinib and siramesine-induced lipid peroxidation and cell death.

### 3.6. Downregulation of HO-1 Combined with the Lysosomotropic Agent Also Induces Cell Death

**Figure 11:**
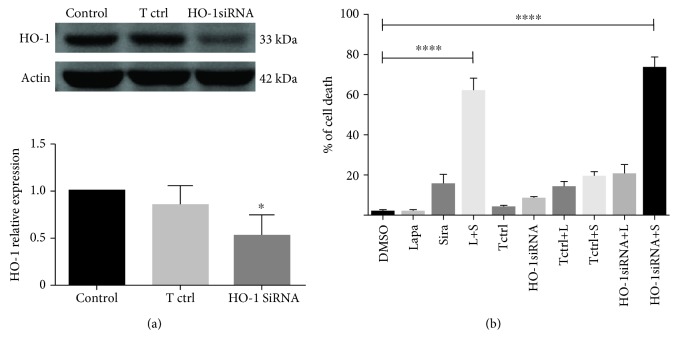
Silencing expression of HO-1 combined with siramesine induces cell death. (a) U87 cells were knockdown in HO-1 expression by siRNA as demonstrated by Western blot; actin was used as a loading control. Densitometry was calculated using ImageJ. Standard error represents three independent experiments (*n* = 3). ∗ represents statistical significance of *P* < 0.05. (b) U87 cells were treated with DMSO, 0.5 *μ*M lapatinib (Lapa), 10 *μ*M siramesine (Sira), or the combination of lapatinib+siramesine (L+S). Transfected (HO-1siRNA) and control transfected (Tctrl) U87 cells were treated with DMSO, 0.5 *μ*M lapatinib (HO-1siRNA+L and Tctrl+L, respectively), and 10 *μ*M siramesine (HO-1siRNA+S and Tctrl+S, respectively) for 24 hours. Cell death was quantified by trypan blue exclusion assay using flow cytometry. Standard error represents three independent experiments (*n* = 3). ∗∗∗∗ represents statistical significance of *P* < 0.0001.

To further verify that HO-1 reduction induced by the combination of siramesine and lapatinib contributes to cell death, we knocked down HO-1 by transfecting U87 cells with a siRNA, resulting in 48% reduction of its expression (Figure 11(a)). We then treated the transfected cells with siramesine or lapatinib and compared the percentage of cell death induced by this combination with the percentage of cell death induced by the lapatinib and siramesine combination. Results showed the same proportion of cell death induced by the combinations HO-1siRNA-siramesine and lapatinib/siramesine with no significant difference between them (Figure 11(b)). This indicates that siramesine in combination with lower HO-1 expression contributes to synergistic cell death.

### 3.7. Inhibition of the Proteasome System Rescues HO-1 Protein Expression

**Figure 12:**
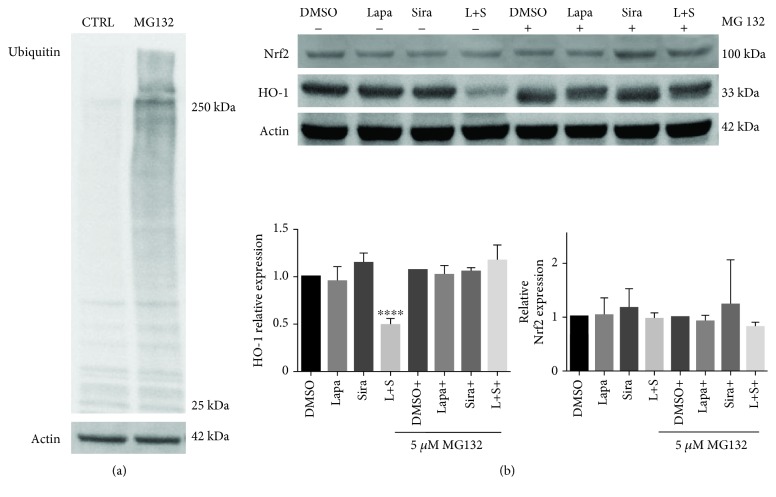
Proteasome inhibition abolish the decrease of HO-1 expression induced by lapatinib-siramesine combination. (a) U87 cells were treated with 5 *μ*M MG132 for 24 hours; then, cells were lysed, Western blot determination of protein ubiquitination using the anti-ubiquitin antibody was performed, and actin was used as a loading control. The image showing protein ubiquitination is representative of three independent experiments (*n* = 3). (b) U87 cells were pretreated with 5 *μ*M MG132 for one hour before treatment with DMSO, 0.5 *μ*M lapatinib (Lapa), 10 *μ*M siramesine (Sira), or the combination of lapatinib+siramesine (L+S). After 24 hours of treatment, cells were lysed, Western blot determination of HO-1 and Nrf2 was performed, and actin was used as a loading control. Densitometry was calculated using ImageJ. Standard error represents three independent experiments (*n* = 3). ∗∗∗∗ represents statistical significance of *P* < 0.0001.

Overexpression of Nrf2 in cancer is related with HO-1 overexpression; therefore, we investigated if the decrease in HO-1 protein levels was a consequence of Nrf2 expression decrease. For this purpose, U87 cells were treated with DMSO, lapatinib and siramesine alone, and in combination and Nrf2 protein expression was analyzed by Western blot. Results showed that there was no significant difference in Nrf2 protein levels in any of the four conditions neither in the whole cell lysates (Fig. [Supplementary-material supplementary-material-1]) nor in the nuclear fraction (Fig. [Supplementary-material supplementary-material-1]). Since the protein levels of the transcription factor Nrf2 were not altered, we next studied the implication of the proteasome system (one of the two principal systems of protein degradation) in HO-1 degradation. The proteasome inhibitor MG132 at a concentration of 5 *μ*M was able to inhibit the proteasome system, at least partially, as shown by the protein ubiquitination (Figure 12(a) and S13). U87 cells were pretreated with 5 *μ*M MG132; the cells were treated with DMSO, 5 *μ*M lapatinib and 10 *μ*M siramesine alone, and in combination, in the presence or absence of MG132. Results showed that proteasome inhibition counteracts the effect of lapatinib and siramesine treatment in HO-1 expression but has no effect in Nrf2 expression (Figure 12(b)). This suggests the decrease in HO-1 expression was due to proteasome degradation and confirms that Nrf2 is not implicated in the regulation of HO-1.

## 4. Discussion

Finding new strategies to induce cell death in hard to treat cancers such as glioblastoma and lung cancer is essential since the classical cell death pathways, such as apoptosis, are actively inhibited in cancer. In this study, we showed that the combination of siramesine, a lysosomotropic agent, with lapatinib, a tyrosine kinase inhibitor, effectively induces synergistic cell death through ferroptosis in both a lung cancer cell line and a glioblastoma cell line. Previous studies in our lab showed that this same combination induced ferroptotic cell death in breast cancer cells; however, the mechanism by which this combination induces cell death was not yet elucidated. We showed that siramesine induced lysosomal membrane permeabilization and the combination of lapatinib and siramesine led to the release of reactive iron and inhibition of the antioxidant systems. This subsequently leads to increased oxidative stress and synergistic ferroptosis.

Siramesine is a known lysosomotropic agent, and we showed an increase LMP following siramesine treatment in U87 cells. Moreover, the combination of siramesine with lapatinib leads to further LMP. This is likely caused not only by the lysosomotropic effect of siramesine but also due to the increased oxidative stress following combinational treatment. Indeed, the accumulation of ROS mediates lysosomal destabilization. It has been observed that lysosomal membrane damage by ROS is the result of intralysosomal iron accumulation that sensitizes lysosomal membrane for disruption [26–28]. We also found that the lapatinib and siramesine combination not only induced LMP but also significantly increased the intracellular amount of iron, as shown by the calcein assay, and that the LMP was followed by the iron increase. These results suggest that siramesine is responsible for LMP, but lapatinib is needed to exacerbate the LMP and induce the release of a significant amount of iron.

Some of the known inducers of ferroptosis, like erastin, act by inhibiting the cystine/glutamate antiporter that is required for GSH synthesis which in turn is needed for the function of glutathione peroxidase 4 (GPX4) thus preventing the accumulation of lipid reactive oxygen species [2, 29, 30]. Recently, it was determined that ferroptosis plays a role in cytotoxic T cell killing of cancer cells [31]. In addition, ferroptosis is regulated by iron transport proteins such as transferrin [32]. We showed that ferroptosis occurs with the combination of siramesine and lapatinib utilizing HO-1 degradation in the U87 glioma cell line. This contributes to the growing amount of methods to induce ferroptosis in cancer cells. HO-1 degradation was also observed in the U373 glioma cell line; in contrast, this was not observed neither in lung cancer cell line A549 nor in breast cancer cell line MDAMB231, raising the hypothesis that this might be a glioblastoma specific mechanism. Further studies in the U373 cell line will be needed in order to establish if HO-1 decrease is involved in the cell death pathway as observed in U87.

HO1 is often upregulated in cancer tissues where it plays a role on carcinogenesis, tumor growth, progression, and metastasis. However, studies have shown contradictory results concerning the role of HO-1 in cancer. On one hand, HO-1 produces by-products biliverdin and bilirubin that are antioxidants and provide protective effects; on the other hand, HO-1 generates iron as a metabolic product of its metabolism and that contribute to cell death. In a study using an I*κ*B*α* inhibitor in breast cancer cells, it was shown that the increase in HO-1 expression activated the ferroptotic pathway through release of iron from degradation of heme and deregulation of iron transport proteins [33]. In another study, it was found that erastin induces expression of HO-1, in which overexpression of this protein accelerated ferroptosis as well as its by-products hemin, CO-releasing molecules. Finally, the inhibition of HO-1 prevented cell death in HT-1080 fibrosarcoma cells [34]. In contrast, studies have shown that although iron released from heme is potentially toxic, ferritin is also upregulated under this conditions as well as cytosolic iron efflux, and both events would resolve the deleterious effect of HO-1 freed reactive iron. One study showed that ultraviolet A irradiation of human skin fibroblasts induced HO-1 expression accompanied by an increased expression of ferritin, which would sequestrate and store the iron released by HO-1 avoiding its participation in the oxidative stress process [35, 36]. Another study that supports this idea showed that iron is first released from heme by the action of HO and then stimulates ferritin synthesis in rat fibroblasts treated with hemin [37]. The role of HO-1 regulating iron efflux was showed by a study where iron efflux was augmented by HO-1 transfection in fibroblasts and reduced in HO-1 knockout mice [38]. In our conditions, it seems that HO-1 has a protective role. We showed that the lapatinib and siramesine combination reduces HO-1 expression and that overexpression of HO-1 not only reversed cell death induced by the combination but it also significantly reduced lipid peroxidation. The effect of HO-1 overexpression in ROS production was modest and this is consistent with another study that showed that GSH and bilirubin have complementary antioxidant activity, where GSH protects water soluble proteins whereas bilirubin protects lipids from oxidation [13]. We also found decreased HO-1 expression following siramesine and lapatinib treatment in glioma cell lines but failed to be decreased in lung and breast cancer cell lines (data not shown). Taken together, the context of ROS generation will dictate the contribution of HO-1 to ferroptosis and might be cell type specific. Understanding this relationship between ROS and HO-1 expression in different cell types will be the focus of future investigation.

In conclusion, lapatinib and siramesine was the most effective tyrosine kinase inhibitor and lysosome disruptor drug combination in inducing synergistic cell death in A549 and U87 cells. This cell death was through ferroptosis mediated by ROS and reduced expression of HO-1 in glioma cells. Further studies will be performed to test the efficacy of the combination of lapatinib with clinical relevant lysosomotropic agents in 3D tumorspheres and in xenograft tumors.

## Data Availability

No data were used to support this study.

## Supplementary Materials

Supplementary Materials Supplementary figure file: a PowerPoint file that contains 13 figures with their corresponding legends.
