# Supplementary material for: Lysosomal Destabilizing Drug Siramesine and the Dual Tyrosine Kinase Inhibitor Lapatinib Induce a Synergistic Ferroptosis through Reduced Heme Oxygenase-1 (HO-1) Levels
Source: Oxid Med Cell Longev. 2019 Sep 17;2019:9561281. doi: 10.1155/2019/9561281 (PMC6766165; doi:10.1155/2019/9561281)
Supplement: Supplementary Materials — Supplementary figure file: a PowerPoint file that contains 13 figures with their corresponding legends. [file 9561281.f1.pdf]

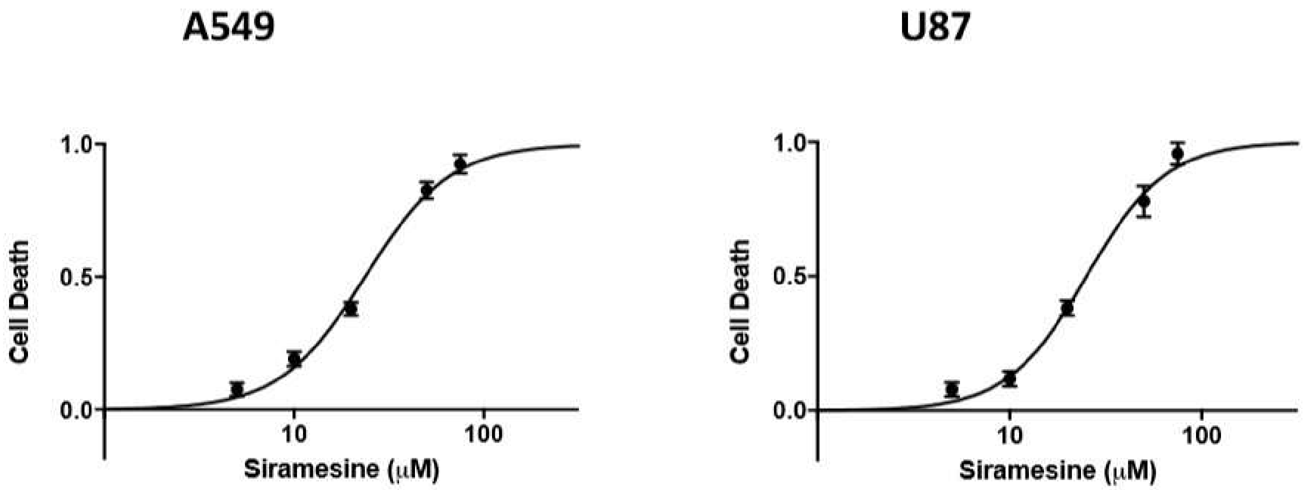

Fig.S1 Siramesine dose response curves. A549 and U87 cells were treated for 24 hours with a range of concentrations of siramesine in order to generate dose-response curves. Cell death was measured by trypan blue staining and quantified by flow cytometry (n=3).

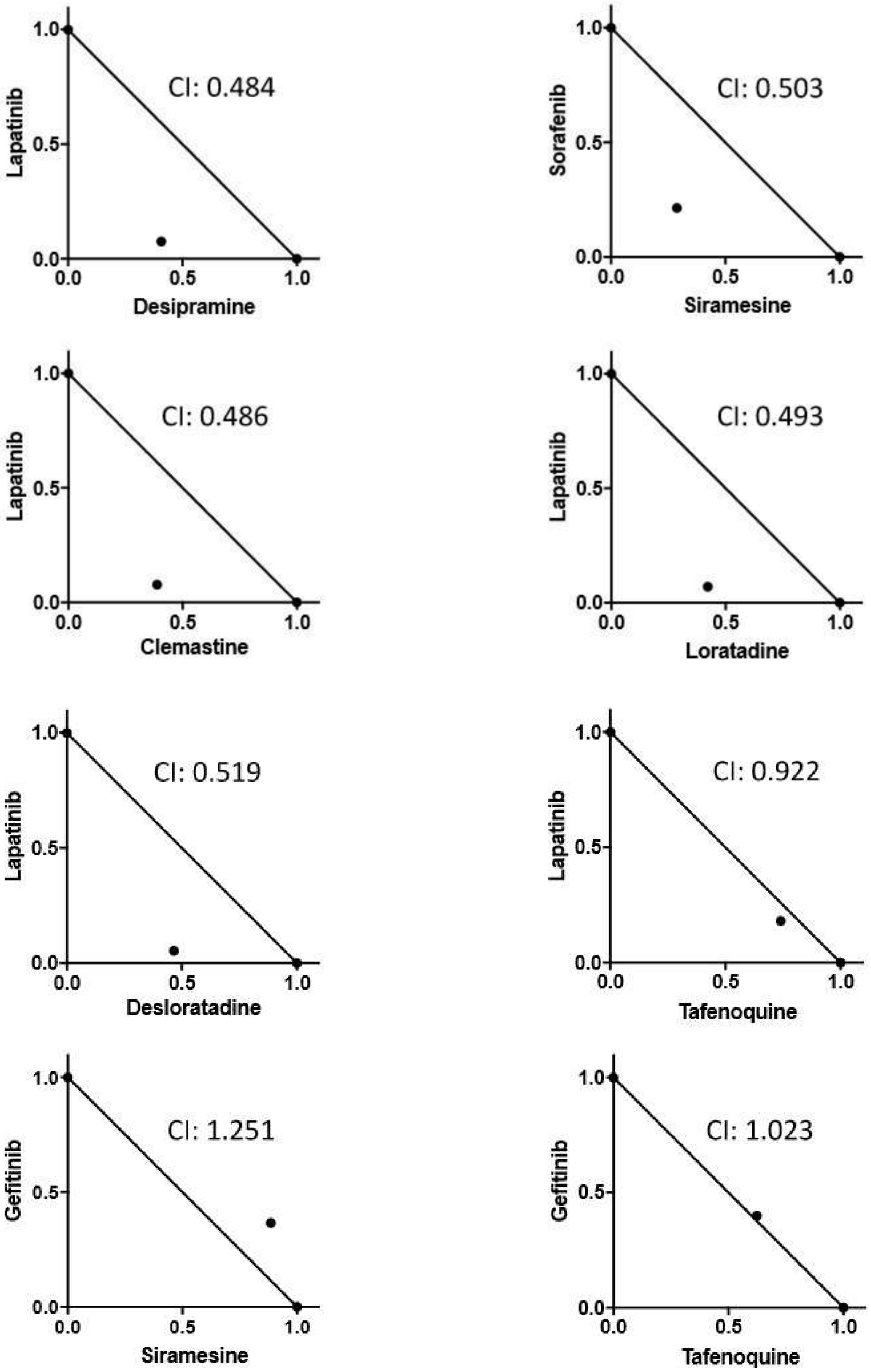

Figure S2: Synergistic cell death is induced by other combinations of lysosome targeting agents and tyrosine kinase inhibitors (TKIs). A549 cells were treated with various combinations of the lysosome targeting agents and TKIs. Isobolograms were generated for the A549 cells to determine whether each combination was synergistic, additive, or antagonistic. Drug-drug interactions were also assessed using the combination index (CI) model. A combination index (CI) <1 indicates synergy, CI=1 indicates that the drugs are additive, and CI>1 indicates the drugs are antagonistic.

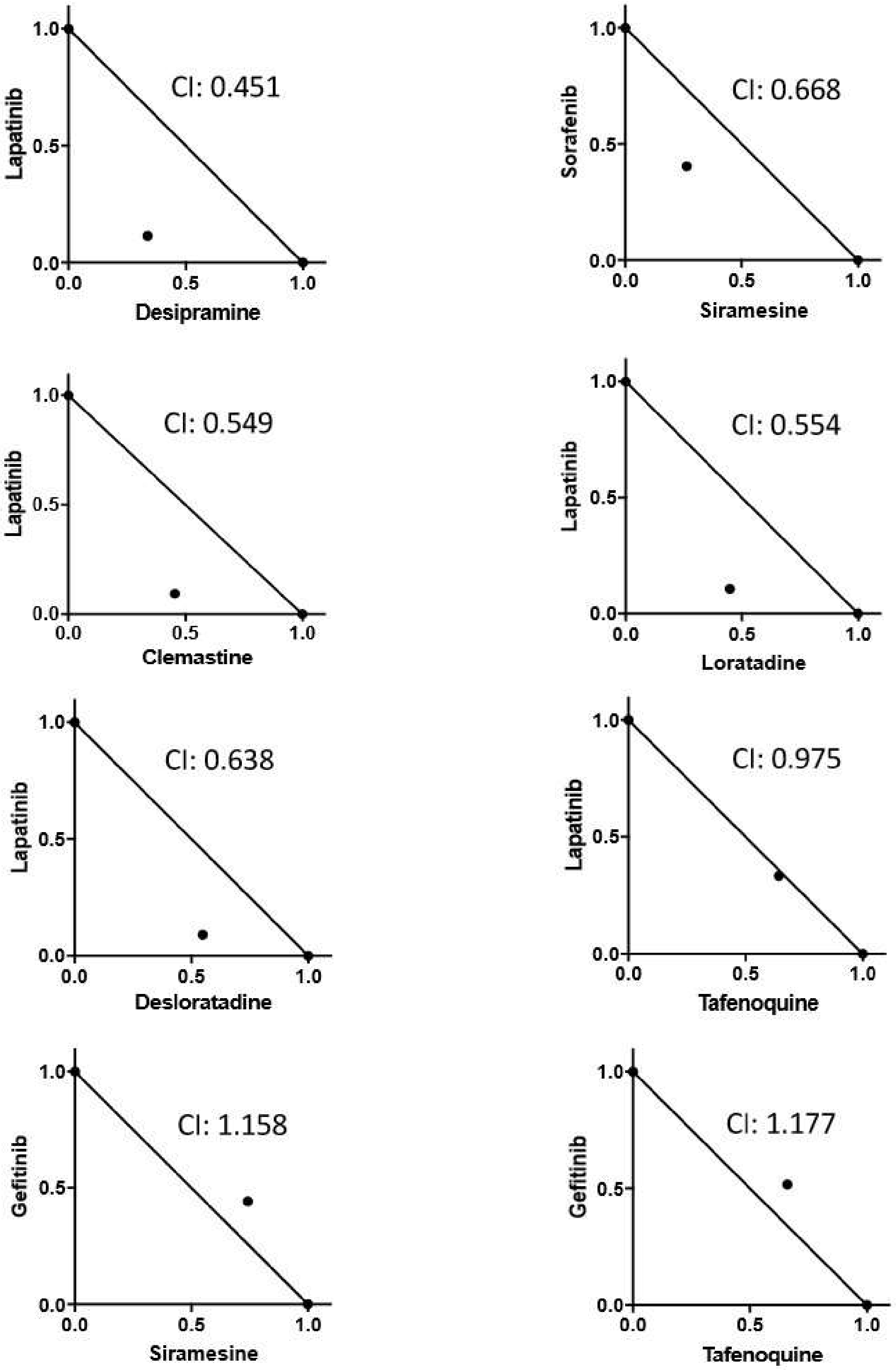

Figure S3: Synergic cell death is induced by other combinations of lysosome targeting agents and tyrosine kinase inhibitors (TKIs). U87 cells were treated with various combinations of the lysosome targeting agents and TKIs. Isobolograms were generated for the U87 cells to determine whether each combination was synergic, additive, or antagonistic. Drug-drug interactions were also assessed using the combination index (CI) model. A combination index (CI) <1 indicates synergy, CI=1 indicates that the drugs are additive, and CI>1 indicates the drugs are antagonistic.

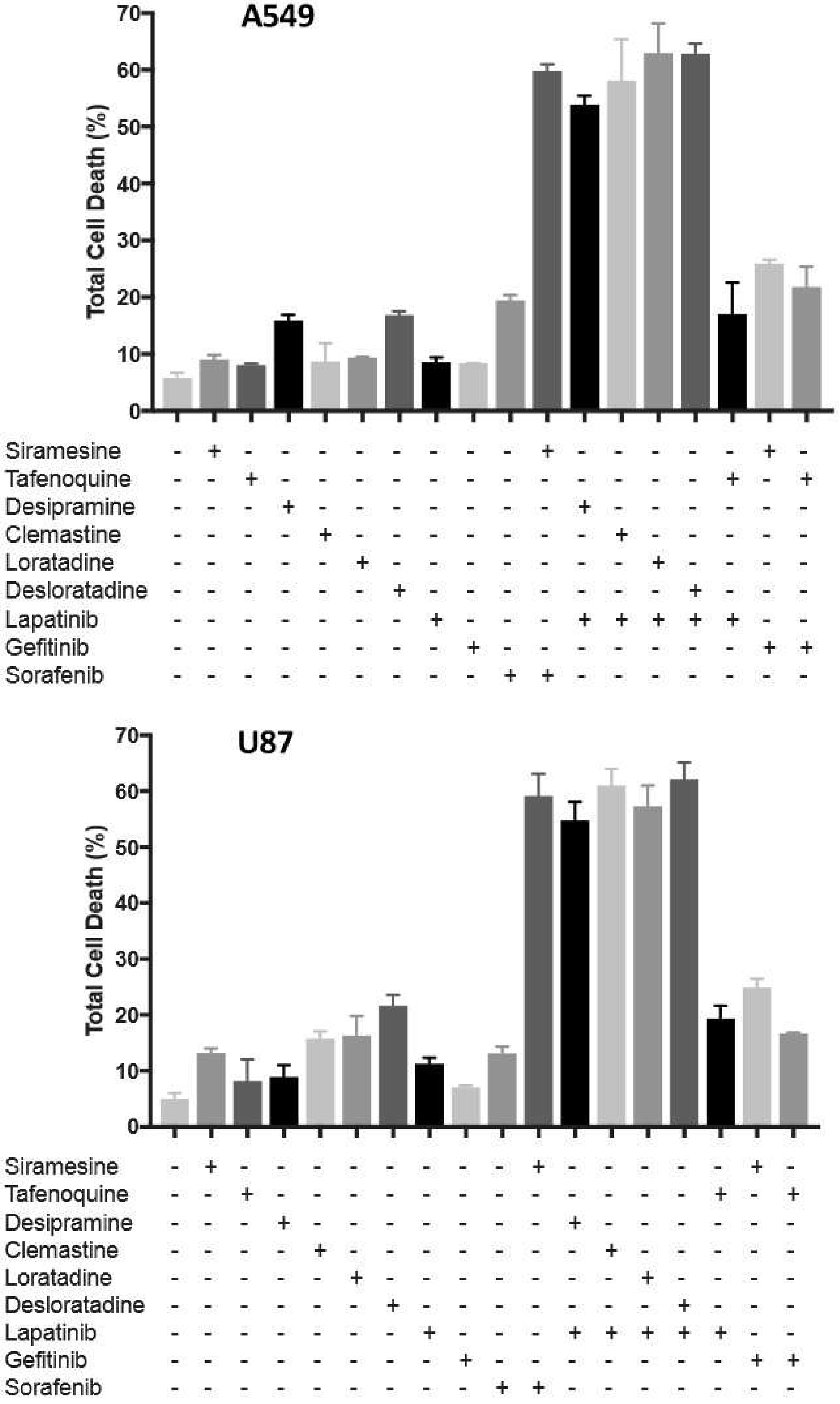

Figure S4: Synergic cell death is induced by other combinations of lysosome targeting agents and tyrosine kinase inhibitors (TKIs). A549 and U87 cells were treated with various combinations of the lysosome targeting agents and TKIs. Cell death was measured by trypan blue staining and quantified by flow cytometry. Each column represents the mean ± 1 SEM (n=3).

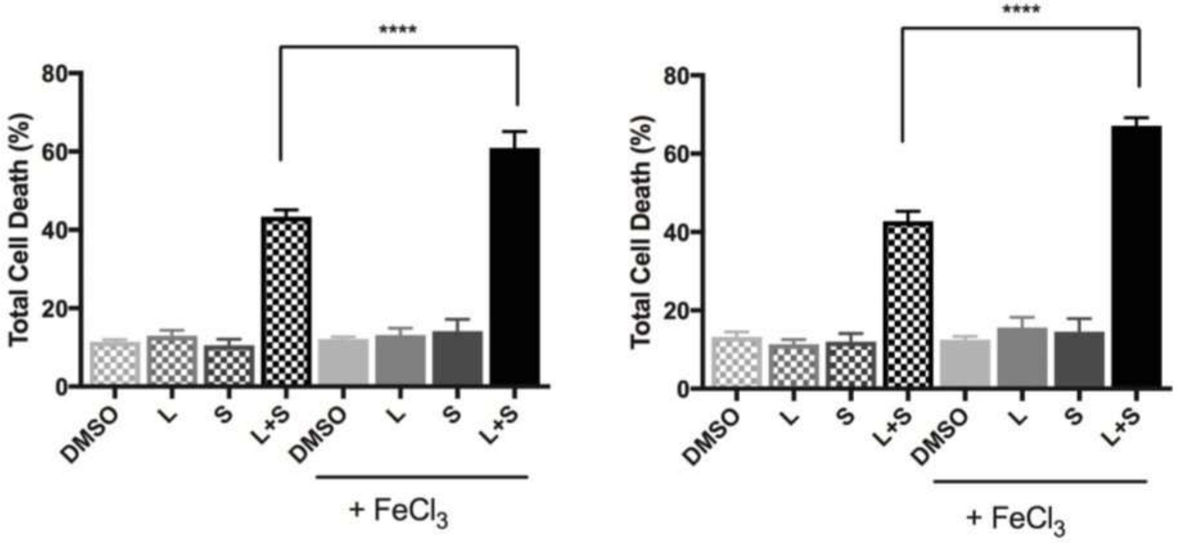

Figure S5: Addition of exogenous iron increased siramesine and lapatinib-induced cell death. A549 and U87 cells were pre-treated with iron chloride (50 µM $FeCl_3$) for one hour before treatment with DMSO, Lapatinib (L), Siramesine (S), or the combination of Lapatinib and Siramesine (L+S) for 24 hours. Cell death was measured by trypan blue staining and quantified by flow cytometry. Standard error represents three independent experiments (n = 3). **** represents statistical significance of P<0.0001.

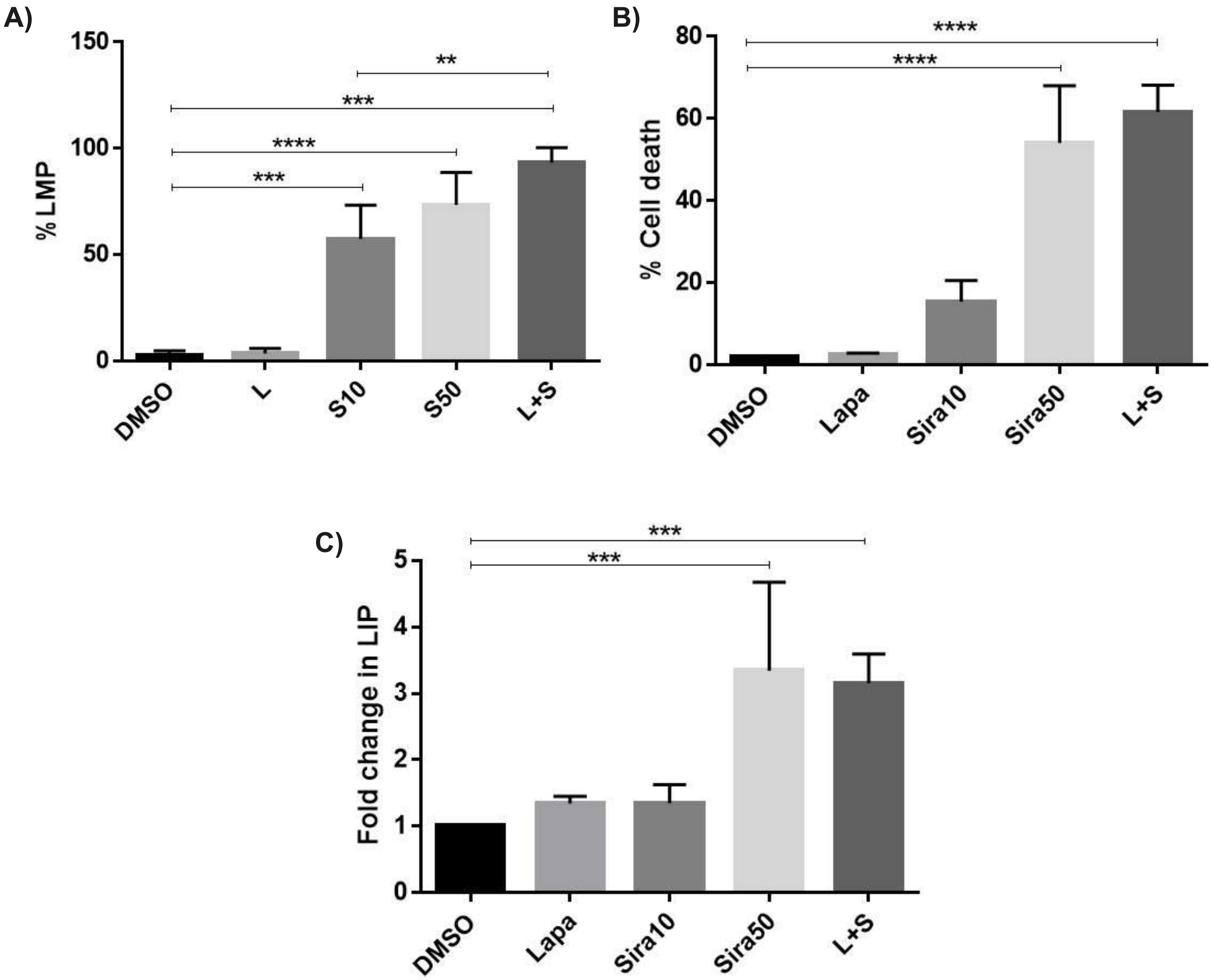

Fig. S6. Higher concentration of siramesine are needed to increase LMP, cell deat and LIP. U87 cells were treated with DMSO, 0.5µM Lapatinib (Lapa), 10µM Siramesin (Sira10) 50µM Siramesine (Sira50) or the combination of Lapatinib + Siramesine (L+S). **A** After four hours treatment cells were incubated with 50nM Lysotracker green for 15 mir LMP was related with the loss of fluorescence intensity of each condition when compare wit the vehicle control fluorescence intensity. **B)** After 24 hours treatment cell death wa measured by trypan blue staining and quantified by flow cytometry. **C)** After 12 hour treatment cells were cells were stained with Calcein-AM (1 µM). Fluorescence signals wer measured by flow cytometry. Standard error represents three independent experiment (n=3). **** represents statistical significance of P<0.0001.

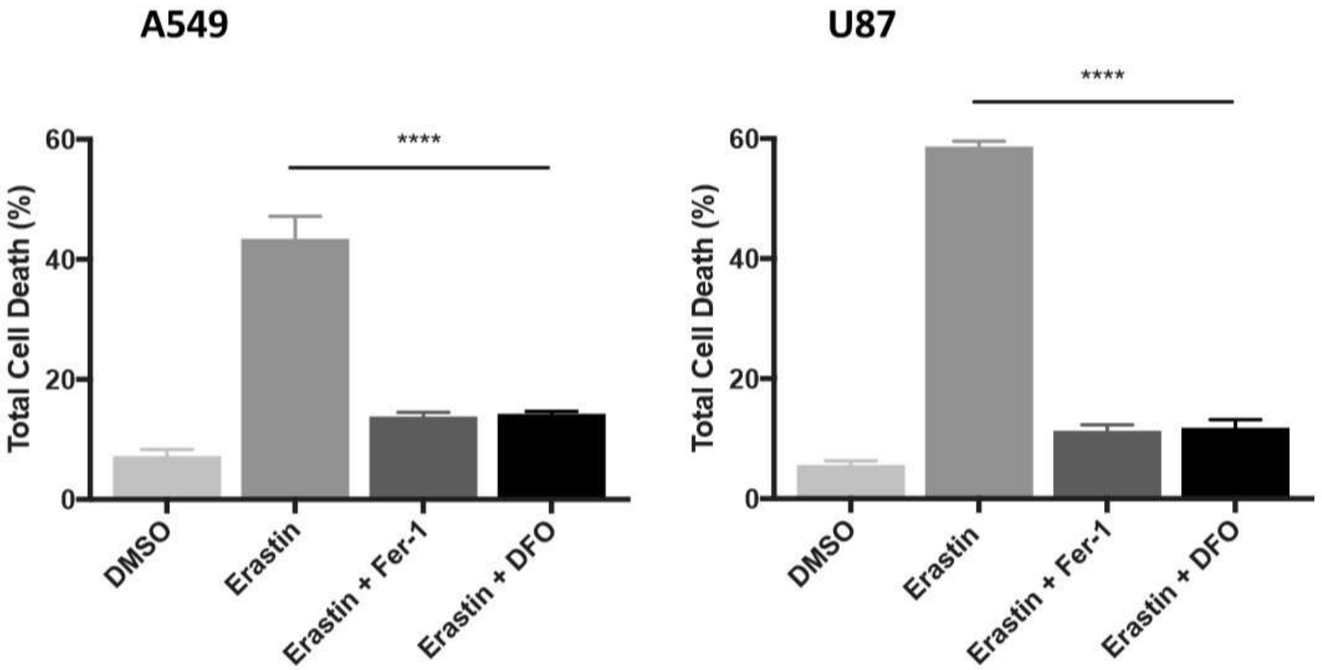

Figure S7: Erastin-induced cell death can be inhibited by ferrostatin-1 (Fer-1) and deferoxamine (DFO). A549 and U87 cells were pretreated with Fer-1 (10 μM) or DFO (200 μM) for one hour before incubation with erastin (15 μM) for 48 hours. Cell death was measured by trypan blue exclusion. Standard error represents three independent experiments (n = 3). **** represents statistical significance of P<0.0001.

A)

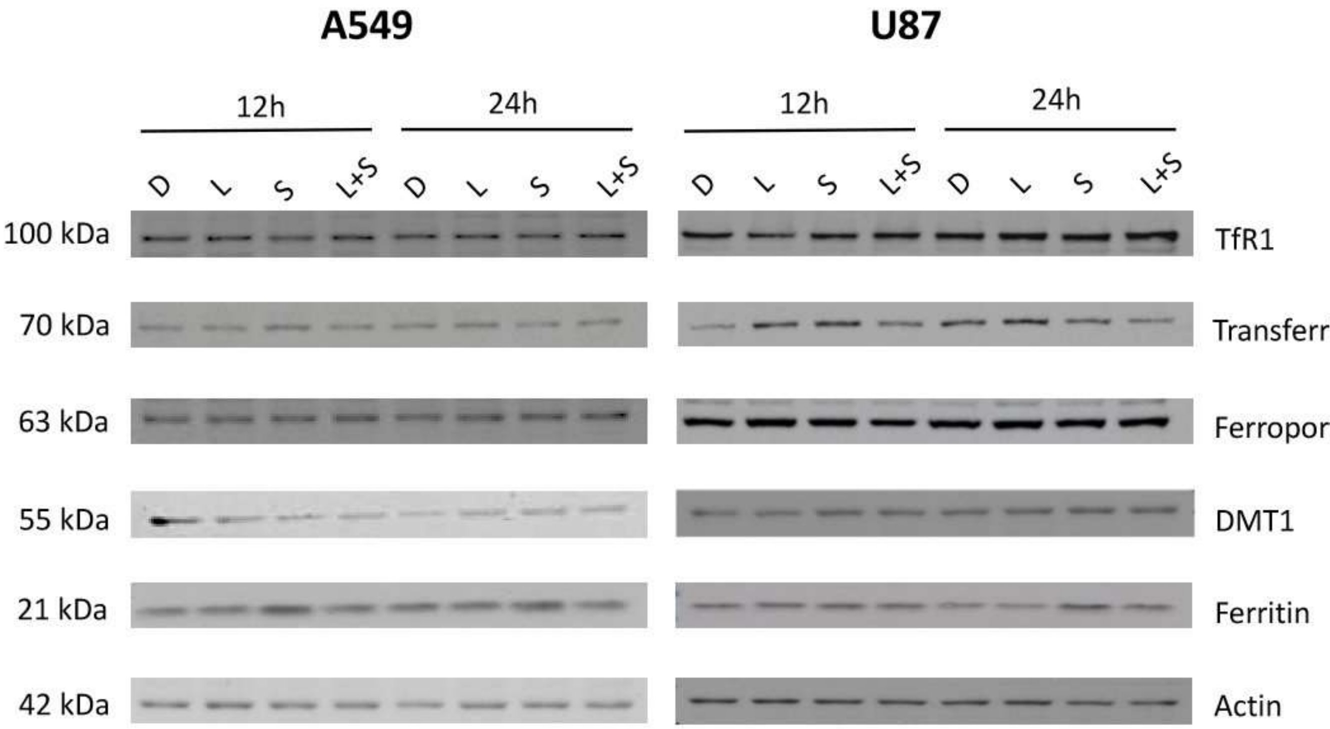

Figure S8: Treatment of A549 and U87 cells with the combination of siramesine and lapatinib does not cause major changes in the levels of iron transport or storage proteins. Western blots were performed on lysates of A549 and U87 cells after 12 hours or 24 hours incubation with DMSO (D), Lapatinib (L), Siramesine (S), or the combination of Lapatinib + Siramesine (L+S). Blots were probed for transferrin receptor 1 (TfR1), transferrin, ferroportin, DMT1 and ferritin. Actin was used as the loading control.

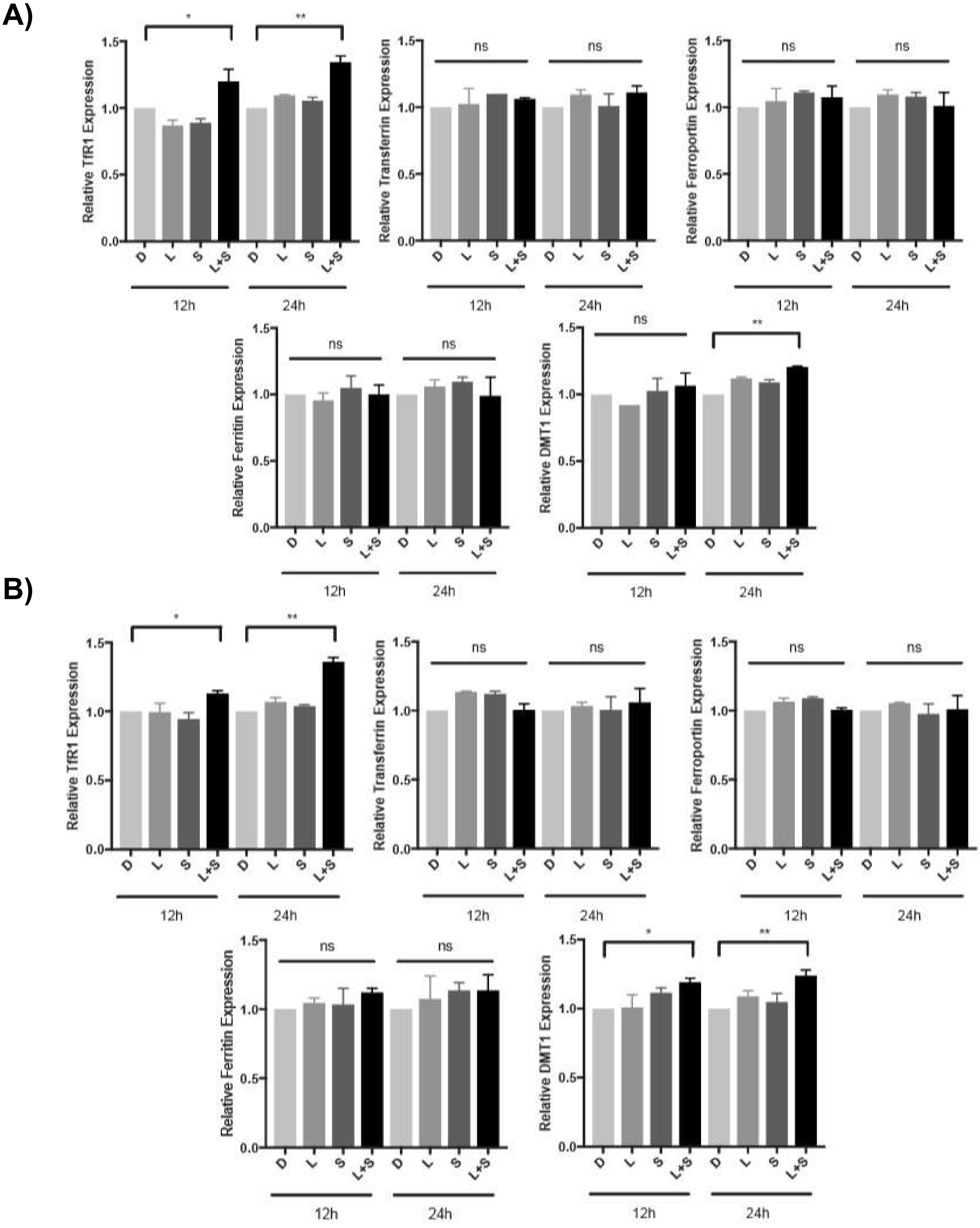

Figure S9: Treatment of A549 and U87 cells with the combination of siramesine an[illegible] lapatinib does not cause major changes in the levels of iron transport or storag[illegible] proteins. Quantitative image analysis was performed on the Western blots from figure S8 fo[illegible] **A)** A549 and **B)** U87 cells. Values obtained for each band of interest were normalized to th[illegible] loading control (actin), and then normalized to the values obtained for DMSO (vehicle control)[illegible] Standard error represents three independent experiments (n = 3); ns, not significant; [illegible] represents statistical significance of P <0.05 and ** represents statistical significance o[illegible] P<0.01).

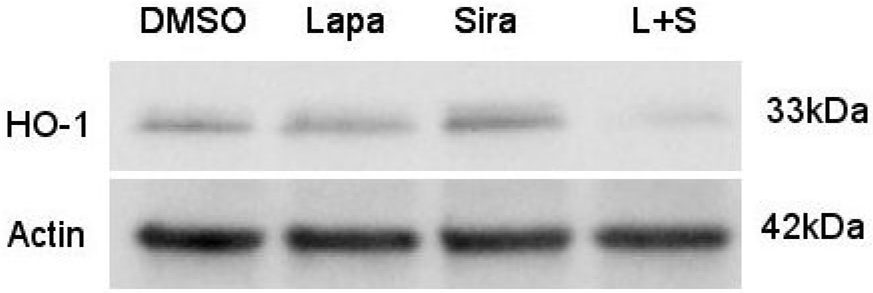

Figure S10: The combination of Lapatinib with Siramesine decreases HO-1 expressio[illegible] in U373 cell line. U373 cells were treated with DMSO, 0.5μM Lapatinib (Lapa), 10μ[illegible] Siramesine (Sira) or the combination of Lapatinib + Siramesine (L+S). After 6 hours treatmen[illegible] cells were lysed and Western blot determination of HO-1 was performed and actin was use[illegible] as a loading control.

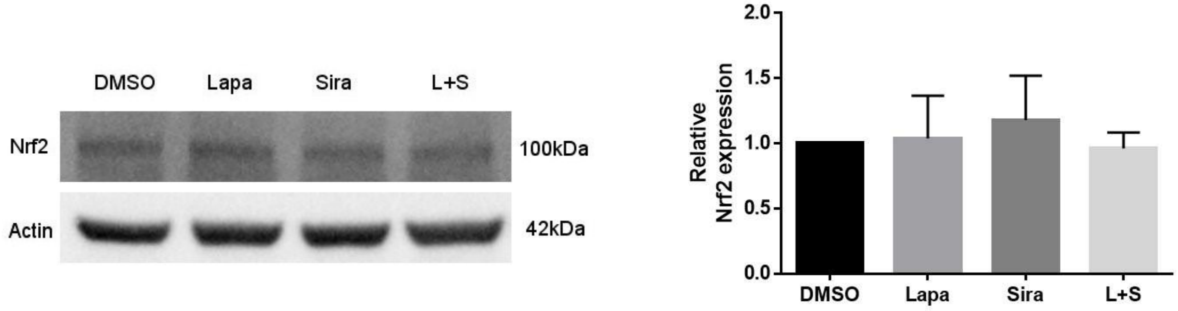

Fig. S11. The combination of Lapatinib with Siramesine fails to change Nrf expression.
U87 cells were treated with DMSO, 0.5μM Lapatinib (Lapa), 10μM Siramesine (Sira) or th combination of Lapatinib + Siramesine (L+S). After 24 hours treatment cells were lysed an Western blot determination of Nrf2 was performed and actin was used as a loading contro Densitometry was calculated using ImageJ. Standard error represents three independer experiments (n = 3).

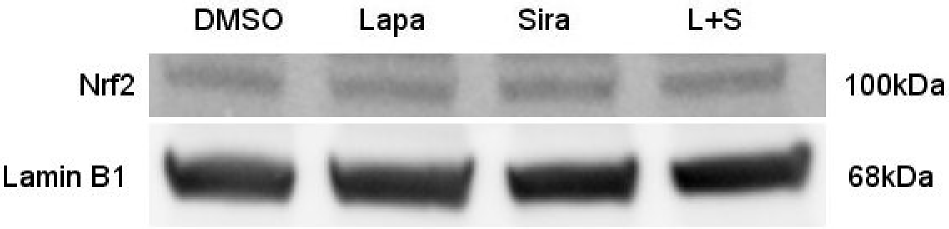

Fig. S12. The combination of Lapatinib with Siramesine fails to change Nrf expression in the nucleous. U87 cells were treated with DMSO, 0.5μM Lapatinib (Lapa 10μM Siramesine (Sira) or the combination of Lapatinib + Siramesine (L+S). After 4 hour treatment nuclear fraction was obtained and Western blot determination of Nrf2 wa performed and lamin B1 was used as a loading control.

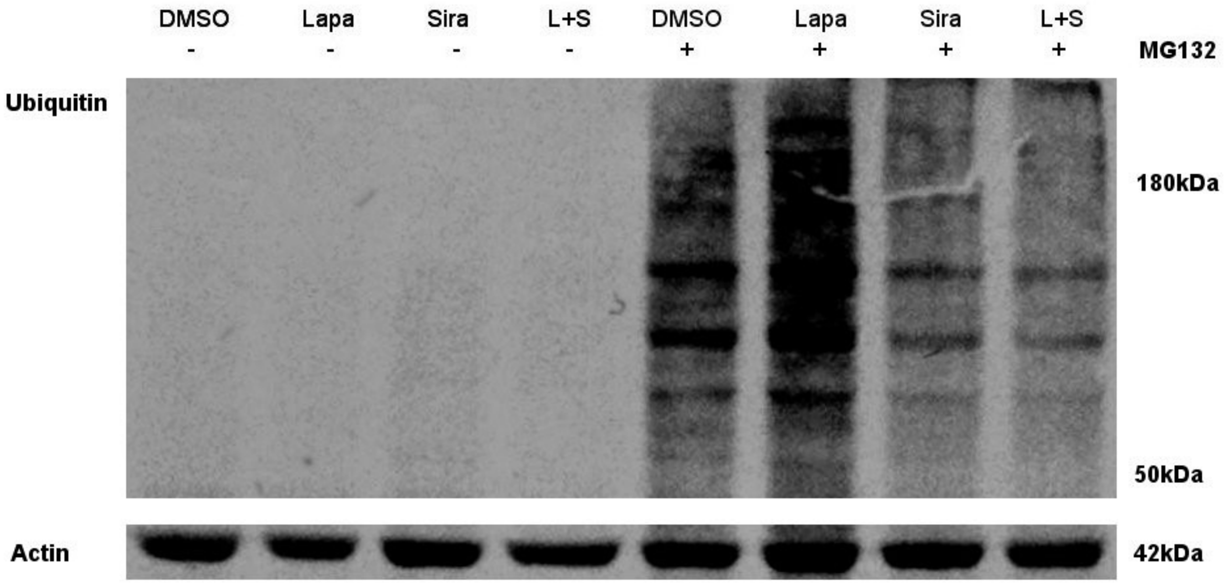

Fig. S13. MG132 effectively inhibits the proteasome activity. U87 cells were pre-treate with 5µM MG132 for one hour before treatment with DMSO, 0.5µM Lapatinib (Lapa), 10µM Siramesine (Sira) or the combination of Lapatinib + Siramesine (L+S). After 24 hour treatment cells were lysed and Western blot determination of protein ubiquitination using a anti-ubiquitin antibody was performed, actin was used as a loading control. The imag showing protein ubiquitination is representative of three independent experiments (n = 3).
